# Retraction

**DOI:** 10.1093/jisesa/iev101

**Published:** 2015-08-07

“Molecular phylogeny and identification of the peach fruit fly, *Bactrocera zonata*, established in Egypt” by Emtithal M. Abd-El-Samie, Zaki A. El Fiky

DOI: http://dx.doi.org/10.1673/031.011.17701

The Journal of Insect Science was notified of concerns about the nucleotide sequence of a gene reported in this paper. Upon further investigation by the Journal, it was determined that the sequence does not translate to a protein and that comparison to other COI gene isolates of *Bactrocera zonata* shows a lack of sequence similarity. In addition, phylogenetic analyses presented in the paper were not appropriate. Furthermore, the paper does not specify that voucher specimens were deposited, and therefore it is not possible to confirm the identification of the Egyptian specimen as *B. zonata*.

The journal has no evidence of intentional author misconduct in sequencing the gene that was isolated. However, based on the issues with the gene sequence and phylogenetic analysis, the Journal of Insect Science believes this paper to be sufficiently flawed that its conclusions cannot be relied upon. Therefore, the journal is retracting this paper.

